# Multisystem Inflammatory Syndrome in Children: A Case Report From Japan

**DOI:** 10.7759/cureus.23682

**Published:** 2022-03-31

**Authors:** Masaki Hisamura, Hikari Asai, Noriyuki Sakata, Hidenori Oi, Hirokazu Taguchi

**Affiliations:** 1 Department of Emergency Medicine, Saitama Medical Center, Saitama Medical University, Kawagoe City, JPN; 2 Department of General Internal Medicine, Saitama Medical Center, Saitama Medical University, Kawagoe City, JPN; 3 Department of Hematology, Saitama Medical Center, Saitama Medical University, Kawagoe City, JPN; 4 Department of Emergency and Critical Care Medicine, Saitama Medical Center, Saitama Medical University, Kawagoe City, JPN

**Keywords:** tafro syndrome, toxic shock syndrome, sars-cov-2, kawasaki disease, japan

## Abstract

This study reports a case of multisystem inflammatory syndrome in children (MIS-C). Although MIS-C is currently not widespread in Japan, it is important to consider this syndrome, particularly when the patient is younger than 21 years and presents with fever and shock symptoms associated with COVID-19. According to the latest statistics updated by the Centers for Disease Control and Prevention in early 2021, the total number of MIS-C patients is only 1659 and there have been no reports from Japan. Therefore, information to accurately diagnose MIS-C is needed. This study is the first case report of MIS-C in Japan, and it proposes information to identify this serious syndrome.

## Introduction

Pediatric patients with coronavirus disease 2019 (COVID-19) can develop a condition that results in a strong inflammatory response affecting multiple organ systems, similar to toxic shock syndrome (TSS) or Kawasaki disease. The first report of this response involved children in England who had to be admitted to the intensive care unit for the multisystem inflammatory syndrome of an unknown cause with features of Kawasaki disease in early May 2020 [1]. Subsequently, similar cases have been reported in Europe and the United States, and it became clear that they were chronologically and geographically related to the outbreak of COVID-19 [2]. These patients tested negative for severe acute respiratory syndrome coronavirus 2 (SARS-CoV-2) on reverse-transcription polymerase chain reaction (PCR) testing but showed positive antibody test results, indicating past infection. Thus, it is believed that the clinical syndrome was due to an inflammatory response after SARS-CoV-2 infection [3]. The World Health Organization (WHO) named this clinical syndrome as multisystem inflammatory syndrome in children (MIS-C). According to the latest statistics updated by the Centers for Disease Control and Prevention in early 2021, the total number of MIS-C patients is only 1659 [4], and to the best of our knowledge, there have been no reports from Japan. Therefore, information regarding the recognition of MIS-C is needed. This study presents the case of an adolescent from Japan who was believed to have MIS-C.

## Case presentation

This case report does not require the consent of the Ethics Committee of our institution and has been anonymized in accordance with the Personal Information Protection Law. The patient's consent was obtained for the publication of this case report.

The patient was a 17-year-old male with a medical history of COVID-19 and no medication history.

History of illness

In August 2020, the patient continued to live with his mother and elder brother, who had contracted COVID-19. He had a fever, which resolved on August 10, 2020. At that time, isolation of COVID-19 patients in Japan was lifted when (1) 10 days had passed since PCR positivity; and (2) the patient had been asymptomatic for 72 hours. The patient’s isolation was lifted on August 16, 2020, when these conditions were met.

On September 5, 2020, the patient became aware of a sore throat and pain in the right neck. On September 6, 2020, he visited a nearby clinic and was prescribed amoxicillin (prescription intent unknown). As the sore throat and right neck pain did not improve, he visited a nearby hospital on September 9, 2020. He was hospitalized following the diagnosis of cervical lymphadenitis, and sulbactam/ampicillin (SAM) was initiated. His blood pressure dropped on the night of admission. He was diagnosed with a septic shock of an unknown fever source, and noradrenaline was started. Antibacterial drugs were changed from SAM to meropenem (MEM) and vancomycin (VAN). On September 11, 2020, chest computed tomography (CT) revealed a large pleural effusion. Thus, acute respiratory distress syndrome was suspected, and tracheal intubation and artificial respiratory management were started. Moreover, an arterial line was inserted into the left radial artery, and a central arterial catheter was inserted into the right inguinal vein. The patient was considered to require advanced treatment and was transferred on the same day to our hospital, which is equipped with intensive care unit (ICU) facilities.

Status at admission

At admission, the patient’s consciousness level was scored E3VTM6 on the Glasgow Coma Scale. His respiratory rate was 16 breaths/minute. Noradrenaline was administered at 0.3 µg/kg/min. His pulse was 104 beats/minute and regular. Further, his blood pressure was 107/84 (96) mmHg, SpO2 was 98% (FiO2 1.0 [100%]), and body temperature was 37.1°C. Regarding physical findings, we observed subconjunctival hemorrhage in both the eyes and tenderness on both sides of the neck.

Blood test findings

His C-reactive protein (CRP) level was elevated (24.51 mg/dL), and his platelet count decreased to 63,000/μL. Myocardial troponin levels were elevated at 305.9 pg/mL. Activated partial thromboplastin time, prothrombin time, and prothrombin time‒international normalized ratio was prolonged to 58.8 s, 14.7 s, and 1.13, respectively, while fibrinogen and D-dimer levels were increased to 574 mg/dL and 5.90 μg/mL, respectively. The SARS-CoV-2 antigen test result was negative.

**Figure 1:**
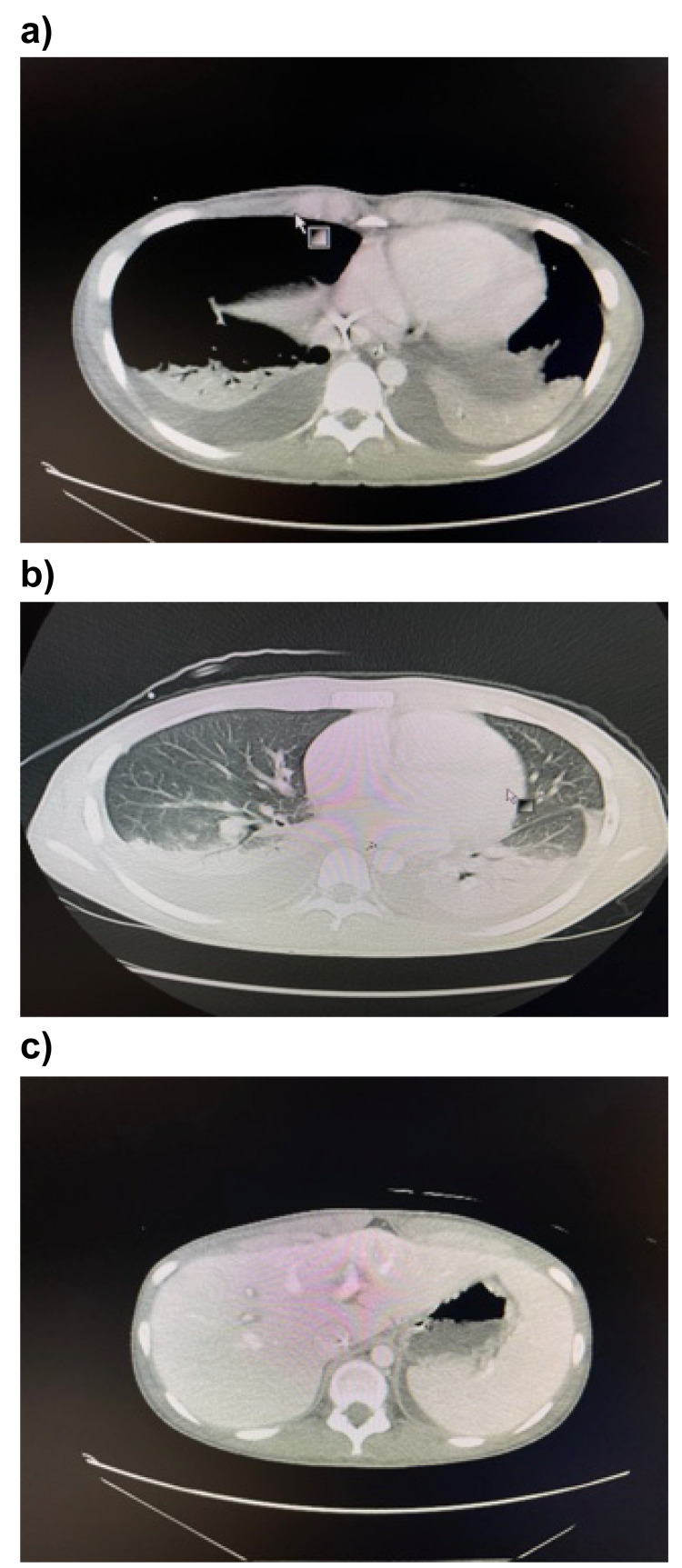
Contrast-enhanced thoracoabdominal computed tomography findings on admission. a) Bilateral pleural effusions. b) Consolidation in both the lungs. c) Splenomegaly.

Infectious endocarditis and septic cardiomyopathy were also suspected; hence, echocardiography was performed. Left ventricular (LV) wall motion showed diffuse mild hypokinesis, LV ejection fraction was 53%, and there was a mild reduction in LV contractility. Thoracoabdominal CT contrast scanning revealed bilateral pleural effusion and splenomegaly (Figure 1).

Hospitalization course

Due to splenomegaly and thrombocytopenia, a type of blood disorder was suspected, and the Department of Hematology was consulted. Based on the findings of splenomegaly, thrombocytopenia, fever, and pleural effusion, TAFRO syndrome [5] was suspected. In addition, there was multiple organ failure after viral infection; thus, the hemophagocytic syndrome was also suspected based on the clinical course. Noradrenaline had to be administered due to hypotension, and because sepsis could not be ruled out, MEM and VAN, which were administered by the previous doctor, were continued, and the patient was transferred to the ICU. On day 2 of the illness, the platelet count improved to 83,000/μL. Body temperature was 39.2°C, and despite noradrenaline use, hypotension persisted with his mean arterial pressure not reaching 65 mmHg. On day 3 of the illness, bone marrow aspiration was performed. The result showed increased myelocytes with toxic granules, and no findings of hematopoietic disorder, blood disorder, or tumor were observed. Platelet count improved to 126,000/μL. He had clear consciousness; hence, we asked him if the tenderness on both sides of his neck had alleviated, and he nodded. Chest tubes were inserted into both sides of the chest for bilateral pleural effusions, with a volume of 200 mL (approximately 100 mL on each side), which can be characterized as exudative.

Regarding the source of fever, the Department of Infectious Diseases was consulted. Lemierre's syndrome was suspected due to pharyngeal pain and neck pain. Lemierre’s syndrome is a disorder characterized by upper respiratory tract infection, followed by internal jugular vein thrombosis, bacteremia, and septic pulmonary embolism. The main symptoms include sore throat, neck pain, and fever. Additionally, the continuation of antibacterial drugs was recommended. On day 4 of the illness, the platelet count improved to 171,000/μL. As the platelet count improved without administering any treatment, this course met the minor diagnostic criteria for TAFRO syndrome; however, no other criteria, except organomegaly, were met; thus, this condition was ruled out. Moreover, in terms of bone marrow pathology, hemophagocytosis, hyperglyceridemia, and hypofibrinogenemia were absent; thus, the hemophagocytic syndrome was also ruled out. No bacteria were detected in the blood cultures. The patient was extubated as he was alert and conscious; nevertheless, hypotension persisted.

On day 6 of the illness, the Department of Infectious Diseases was consulted again. Lemierre's syndrome was ruled out as no bacteria were detected in the blood cultures, and contrast-enhanced CT showed no thrombosis. However, since the history of SARS-CoV-2 infection, the patient’s age, and the persistent fever strongly matched the diagnostic criteria of MIS-C, this condition was considered to be highly likely.

**Figure 2:**
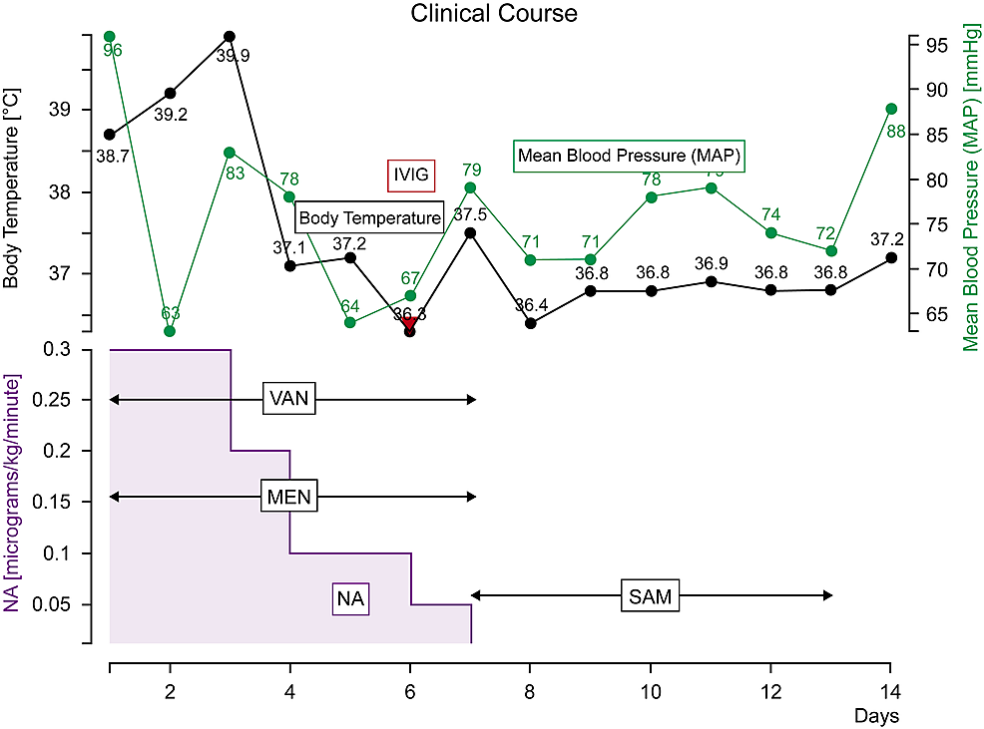
Clinical course of the patient SAM: sulbactam/ampicillin; MEM: meropenem; NA: noradrenaline; IVIG: intravenous immunoglobulin; VAN: vancomycin

Being the first facility to treat MIS-C in Japan, a definitive diagnosis could not be established. Thus, MEM and VAN were discontinued and changed to SAM, considering the possibility of Lemierre's syndrome. Intravenous immunoglobulin therapy (IVIG) was started at 2 g/kg as MIS-C was suspected. From this point, the mean arterial blood pressure constantly exceeded 65 mmHg, and noradrenaline was gradually reduced. On day 7 of the illness, noradrenaline was withdrawn, and the arterial line and central venous line were removed. Pleural effusion drainage was stopped, and the chest tubes were removed. On day 8 of the illness, the patient was transferred from the ICU to the general ward. The patient demonstrated a good clinical course after the transfer and was definitively diagnosed with MIS-C based on the clinical course. On day 13 of the illness, SAM was also discontinued. On day 14 of the illness, the patient was discharged (Figure 2). Currently, he visits the hospital once every two weeks; however, his progress has been good, and he could perform daily life activities without any difficulty.

## Discussion

The patient, in this case, initially exhibited neck pain, fever, hypotension, and pleural effusion. Treatment by antibacterial drugs was started with empiric antimicrobial therapy, following the diagnosis of septic shock with an unknown source of fever, and the patient was considered to be in need of advanced medical care and was transferred to our hospital. Initially, TAFRO syndrome [5] and the hemophagocytic syndrome were also suspected; however, they were ruled out mainly because the platelet count increased during follow-up. Lemierre’s syndrome was also ruled out based on symptomatic treatment, and noradrenaline was administered to treat hypotension. Although the source of infection was unknown, antibacterial drugs were continued. Tachycardia, impaired consciousness, and livedo reticularis, which are usually noted in patients with sepsis, were not observed. Hypotension did not improve despite the administration of antibacterial and antihypotensive drugs, and there was no impression of distributive shock caused by infection. The Department of Infectious Diseases was consulted, and due to a history of SARS-CoV-2 infection within the previous four weeks, MIS-C was suspected. As the diagnostic criteria of MIS-C were met [6], the patient was treated for MIS-C. After initiating IVIG, his blood pressure increased; therefore, the antihypotensive drug could be withdrawn, all infusion lines removed, and the patient discharged.

To the best of our knowledge, there had been no reported case of MIS-C in Japan (as of November 2021), and it is not well known, even in the medical setting. Thus, there was a slight delay in the diagnosis of this patient. Consequently, education and awareness on MIS-C are necessary.

There are two possible reasons for the delayed diagnosis in this case. First, in Japan, there was no increase in the number of cases similar to Kawasaki disease associated with SARS-CoV-2, unlike in Europe and the United States; thus, MIS-C was not considered initially. In Japan, questionnaire surveys on the relationship between Kawasaki disease and SARS-CoV-2 have been conducted twice by the Japanese Society of Kawasaki Disease. The first survey investigated the situation from February to April 2020, a period that included the first wave of the COVID-19 pandemic in Japan. Consequently, it has been reported that the incidence of Kawasaki disease and that of severe Kawasaki disease were about the same as, or tended to be less than, those in several annual reports before January 2020, and no case of COVID-19 complicated with Kawasaki disease had been confirmed. The second survey investigated the situation from May to October 2020, a period that included the second wave of the COVID-19 pandemic. Thus, it has been reported that the number of hospitalized patients with Kawasaki disease was reduced by half as compared to the previous year, and overall, 2 of 99 patients with Kawasaki disease tested positive for SARS-CoV-2; this rate was not significantly different from the SARS-CoV-2-positive rate in children hospitalized for conditions other than Kawasaki disease [7].

The other reason is the lack of specific symptoms of MIS-C. The diagnostic criteria may also occur in sepsis. Furthermore, MIS-C is characterized by digestive symptoms, such as diarrhea and vomiting, and abnormal cardiac function with cardiogenic shock [8]; however, no digestive symptoms were observed in our case. Moreover, in this case, blood disorder was also suspected due to splenomegaly and thrombocytopenia; however, these features are not included in the diagnostic criteria of MIS-C. Therefore, as MIS-C has various non-specific symptoms, it can only be diagnosed based on reliable, currently available information. A precondition of MIS-C is past infection with SARS-CoV-2. Furthermore, the frequency of fever is 100%, and hypotension is also common at an incidence of 64.7%, followed by shock at 58.8% [9]. Symptoms resembling Kawasaki disease, such as rash, conjunctivitis, swelling of the four limbs, changes in the oral mucosa, and cervical lymphadenopathy, are common at an incidence of 70.6% [9]; however, most medical professionals treating adults, in particular, have no experience with Kawasaki disease. Consequently, it may be difficult to identify these features as symptoms suggestive of MIS-C. The patient, in this case, had cervical lymphadenopathy; however, no medical professional at the previous hospital or our hospital regarded this as a symptom resembling Kawasaki disease. Combining these with the diagnostic criteria of MIS-C established by the WHO and the Centers for Disease Control of the United States [6,8], when a patient aged below 21 years, who has a history of infection with SARS‒CoV‒2 within the previous four weeks, has a fever that persists for more than 24 hours and presents with hypotension and shock symptoms, only MIS-C can be considered likely. Diseases with clinical symptoms similar to those of MIS-C that require special attention during differentiation include Kawasaki disease, TSS, and secondary hemophagocytic lymphohistiocytosis (SHLH) [3].

Kawasaki disease is characterized by acute vasculitis, with the main lesions occurring in the coronary arteries, and it mainly develops in early childhood. Additionally, it is characterized by rash, cervical lymphadenopathy, and changes in the ocular and oral mucosa [10]. In addition, Kawasaki disease tends to present with neutrophil-dominant leukocytosis and thrombocytosis. However, thrombocytopenia, as in this case, is rare in Kawasaki disease [3]. In addition, in terms of cardiac findings, Kawasaki disease rarely presents with abnormal cardiac function and hypotension [11], as observed in this case; thus, it was ruled out.

TSS is a condition that develops when “superantigens,” which are proteins that non-selectively stimulate T cells, uncontrollably activate the immune system, releasing large amounts of cytokines. Bacterial species, such as Staphylococcus aureus and Streptococcus, are known to produce exotoxins that function as superantigens [3]. Its clinical symptoms include hypotension, erythroderma zoster, mucosal lesions, renal dysfunction, hepatic dysfunction, and pancreatic dysfunction [12], which are similar to the clinical symptoms of MIS-C. It is treated by administering antibacterial drugs to treat the causative infection, and IVIG is sometimes administered for hypotension in refractory cases [13]. In the present case, TSS was ruled out because the patient developed no infection, and neither erythroderma zoster nor mucosal lesion was observed.

Moreover, SHLH induces a cytokine storm via a strong immune response. Macrophage lineage cells are activated by excess cytokines phagocytose blood cells of every lineage, inducing pancytopenia [14]. Primary hemophagocytic lymphohistiocytosis is caused by abnormalities in genes controlling the degranulation of natural killer cells and cytotoxic CD8+ lymphocytes [14,15]. SHLH can be caused by drugs, malignant tumors, and infections. In particular, viral infection is well known as a trigger of SHLH [16]. Autoimmune diseases may also trigger this condition, which is also referred to as macrophage activation syndrome [17]. Besides decreased leukocyte count, thrombocytopenia, decreased erythrocyte sedimentation rate, and systemic inflammation, including high CRP levels, hypertriglyceridemia, and elevated D-dimer levels, SHLH also presents with organ dysfunction, such as coagulopathy, liver failure, central nervous system disorder, and cardiac dysfunction [18]. However, SHLH was ruled out in this case, as the patient did not experience hypertriglyceridemia, and his platelet count improved naturally.

There is no clear guideline for treating MIS-C [3]. In addition to symptomatic therapy, the use of IVIG is desirable. IVIG has a marked advantage, as it is also used when Kawasaki disease, TSS, and SHLH cannot be ruled out [3]. In this case, from the implementation of IVIG treatment, both hypotension and the clinical course improved. However, IVIG was administered for six days, and the delayed diagnosis could have negatively impacted the disease outcome.

## Conclusions

MIS-C is a delayed immune response to SARS-CoV-2, according to a previous report. Therefore, the number of MIS-C cases will certainly increase in the future. Moreover, MIS-C often requires resuscitation due to circulatory collapse; therefore, this disease should be widely recognized by clinicians. As MIS-C is still a rare disease in Japan, it is difficult to recognize. Our case study illustrates the importance of suspecting MIS-C when attending to patients younger than 21 years presenting with fever and shock symptoms associated with a previous COVID-19 episode.
